# Supplementary material for: Cytosolic S100A8/A9 promotes Ca2+ supply at LFA-1 adhesion clusters during neutrophil recruitment
Source: eLife. 2024 Dec 19;13:RP96810. doi: 10.7554/eLife.96810 (PMC11658764; doi:10.7554/eLife.96810)
Supplement: Figure 3—source data 1. — Original membranes corresponding to Figure 3H and I. paxillin, p-paxillin, Pyk2, and p-Pyk2 membranes are depicted and representative blots were then cropped and edited. Rectangle boxes indicate the representative bands used in the figure. Lowest membranes were cut before the staining to save staining solution and fit more membranes at the same time. GAPDH was always employed as an internal control. Chamaleon Duo Pre-stained Protein Ladder was used as molecular weight marker. [file elife-96810-fig3-data1.zip › p-Pyk2_Paxillin WB.pdf]

p-Pyk2

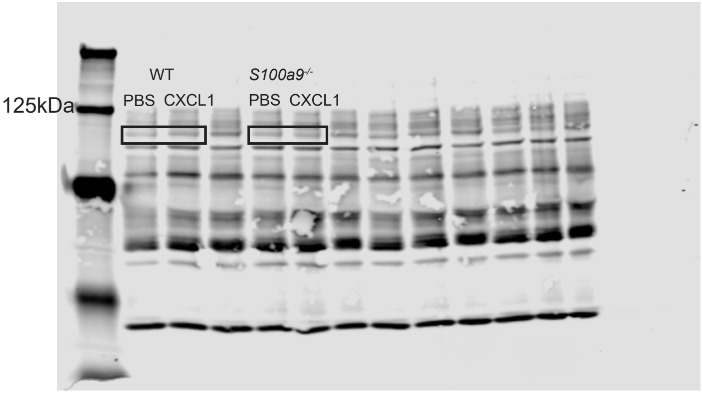

tot Pyk2

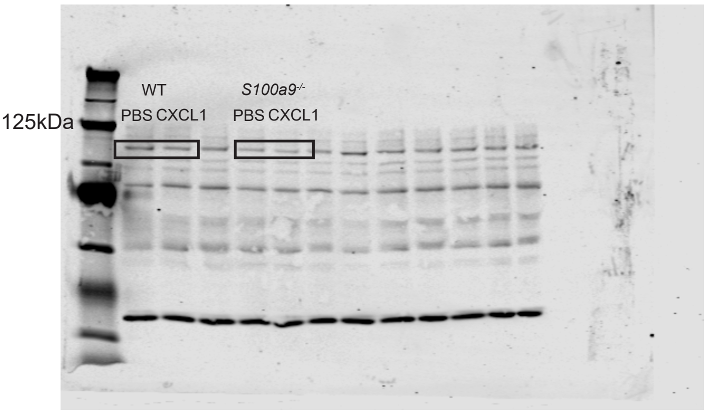

p-Paxillin

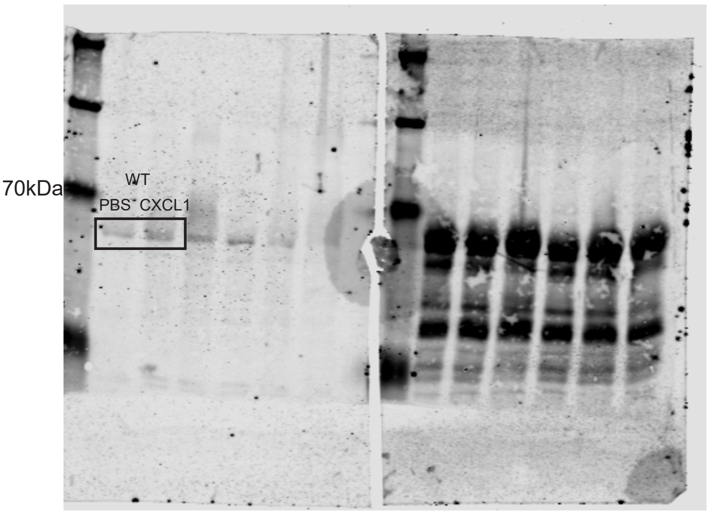

p-Paxillin + tot Paxillin

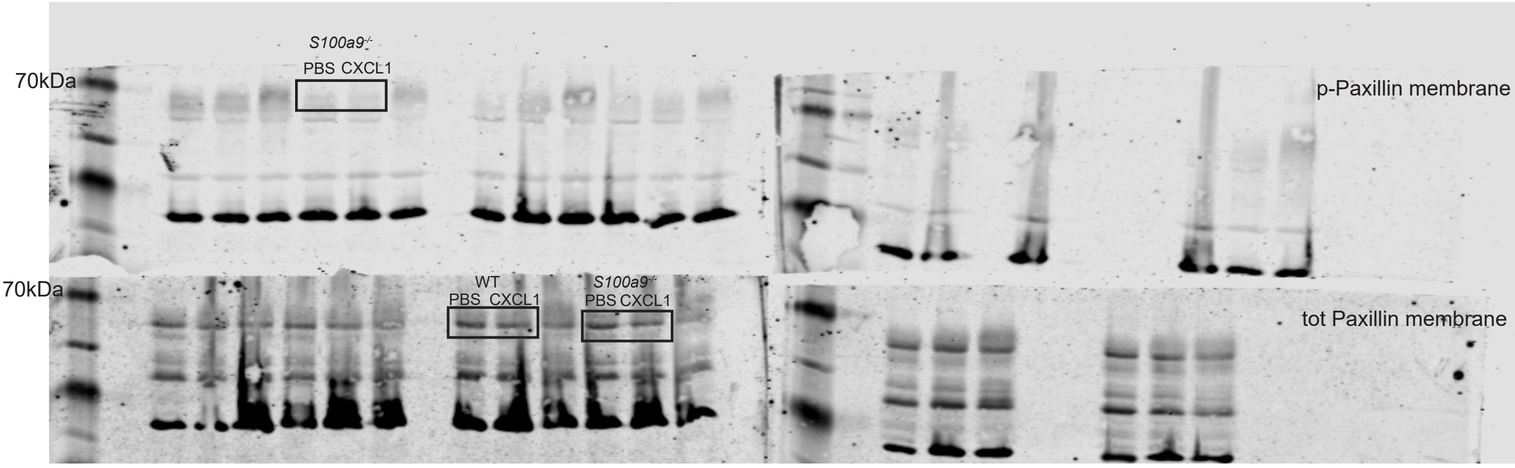

Figure 3-source data 1. Original Membranes corresponding to Figure 3, panel H and I. Paxillin, p-Paxillin, Pyk2 and p-Pyk2 membranes are depicted and representative blots where then cropped and edited. Lowest membranes were cut before the staining to save staining solution and fit more membranes at the same time. GAPDH was always eployed as an internal control. Chamaleon Duo Pre-stained Protein Ladder was used as molecular weight marker.
