# Supplementary figures and images for: Cytosolic S100A8/A9 promotes Ca2+ supply at LFA-1 adhesion clusters during neutrophil recruitment
Source: eLife. 2024 Dec 19;13:RP96810. doi: 10.7554/eLife.96810 (PMC11658764; doi:10.7554/eLife.96810)

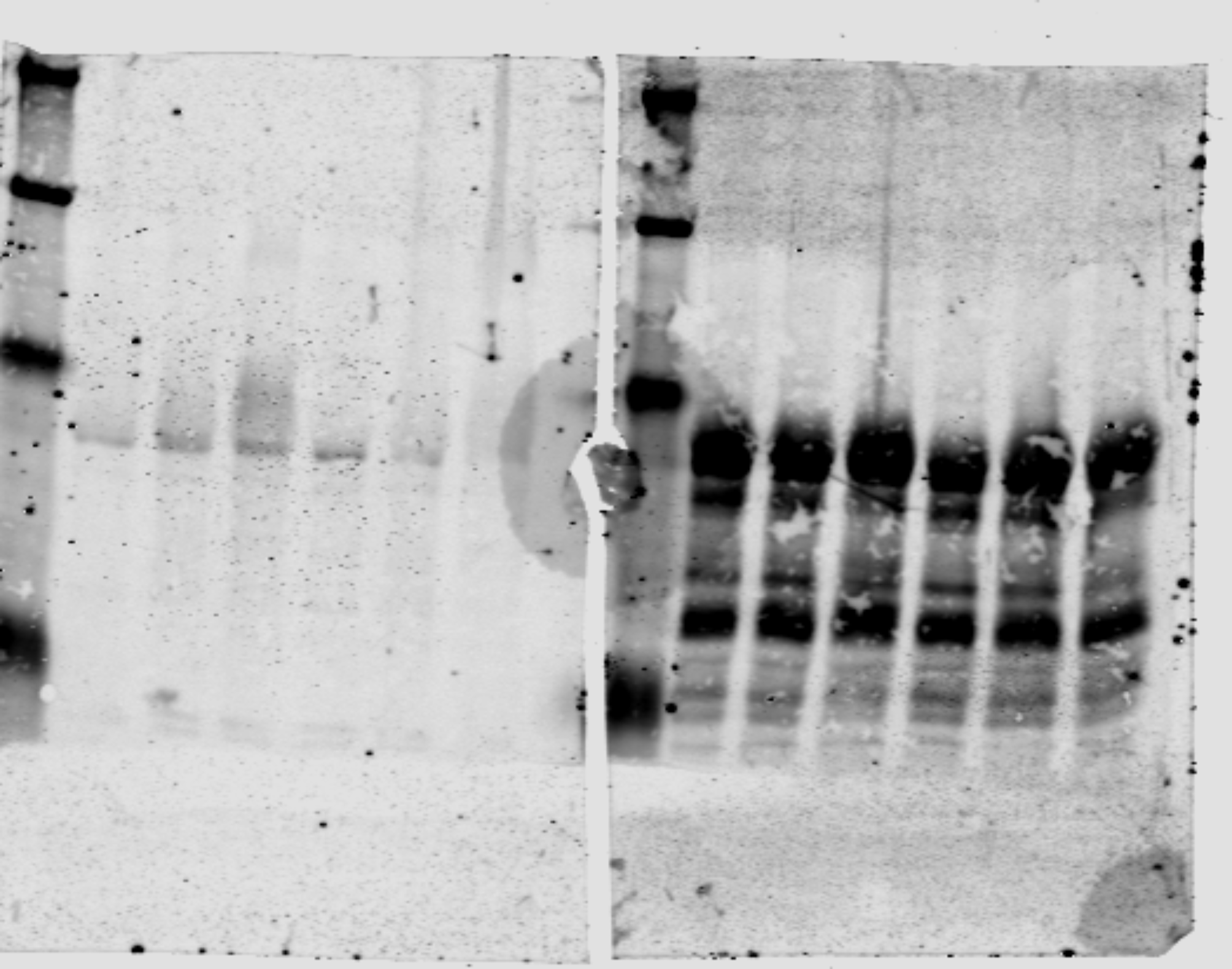

Supplement: Figure 3—source data 2. — Original membranes corresponding to Figure 3H and I. Paxillin, p-paxillin, Pyk2, and p-Pyk2 original membranes. [file elife-96810-fig3-data2.zip › p-Paxillin uncropped blot_WT.tif]

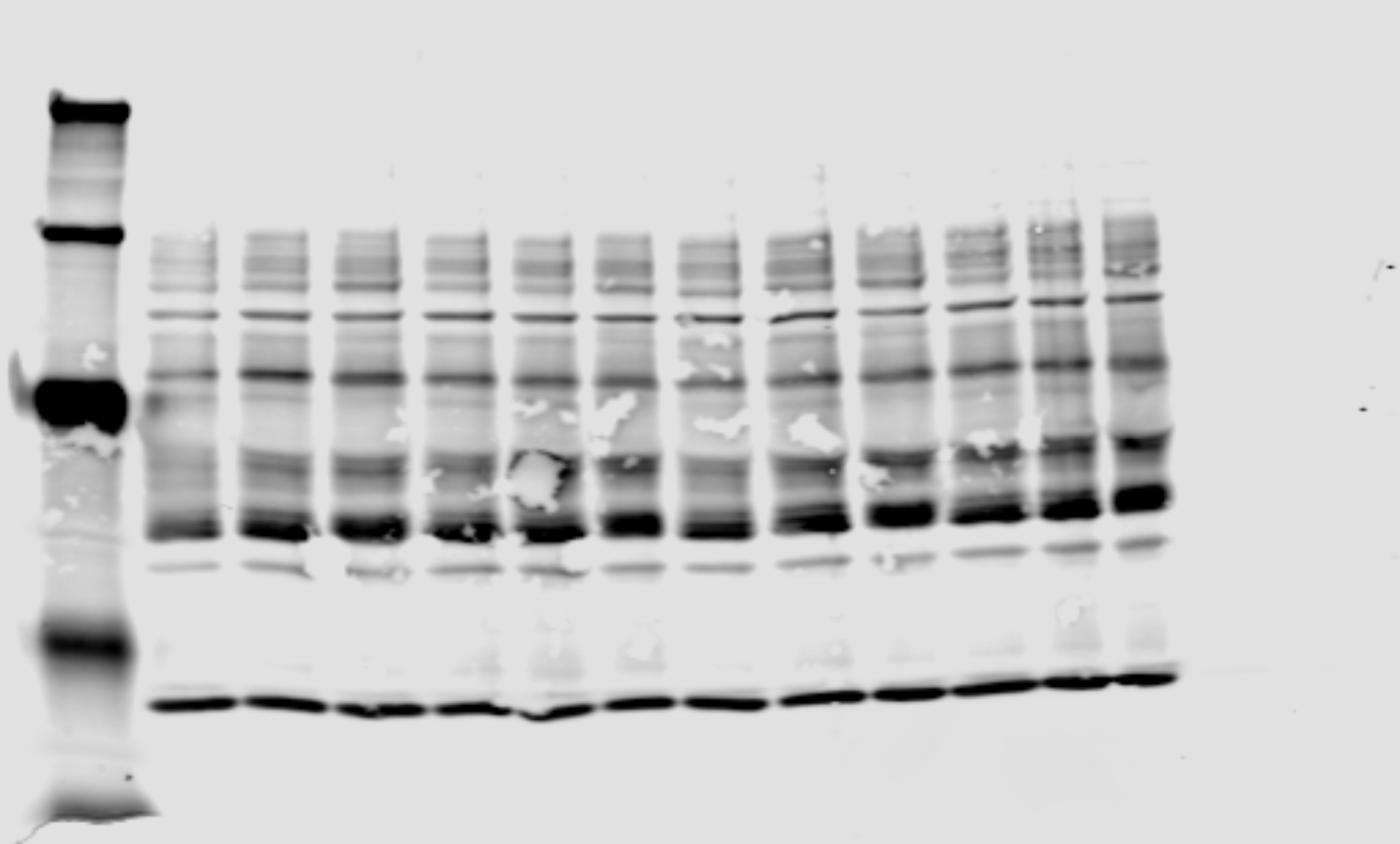

Supplement: Figure 3—source data 2. — Original membranes corresponding to Figure 3H and I. Paxillin, p-paxillin, Pyk2, and p-Pyk2 original membranes. [file elife-96810-fig3-data2.zip › p-Pyk2 uncropped blot.tif.tif]

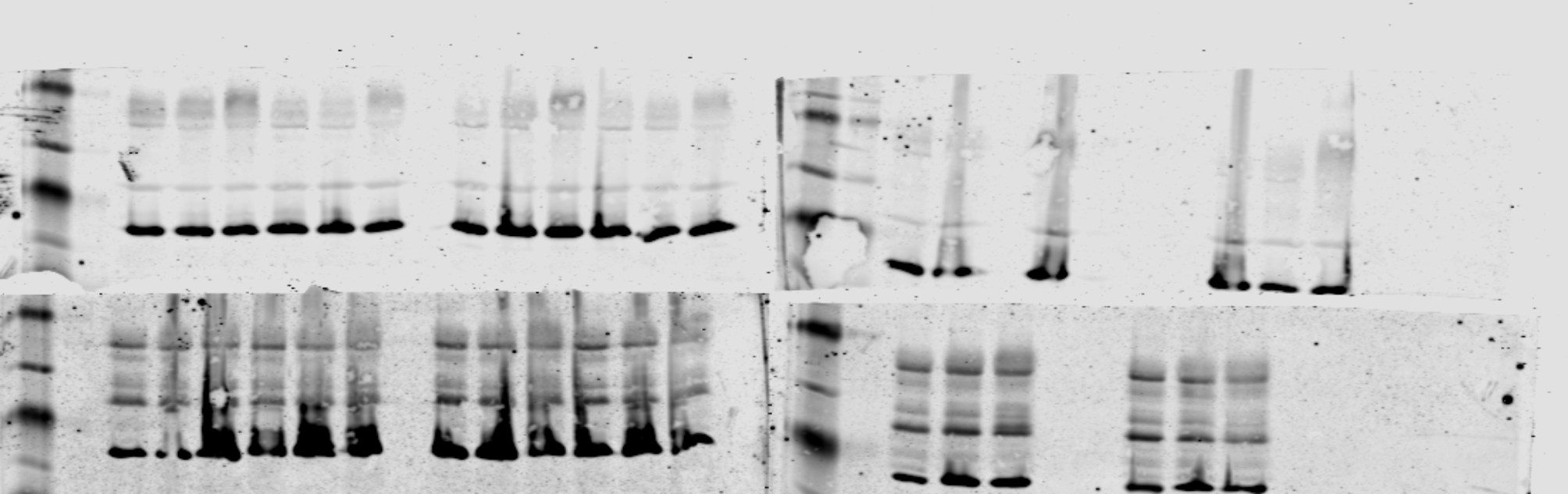

Supplement: Figure 3—source data 2. — Original membranes corresponding to Figure 3H and I. Paxillin, p-paxillin, Pyk2, and p-Pyk2 original membranes. [file elife-96810-fig3-data2.zip › tot Paxillin and p-Paxillin S100 uncropped blot.tif.tif.tif]

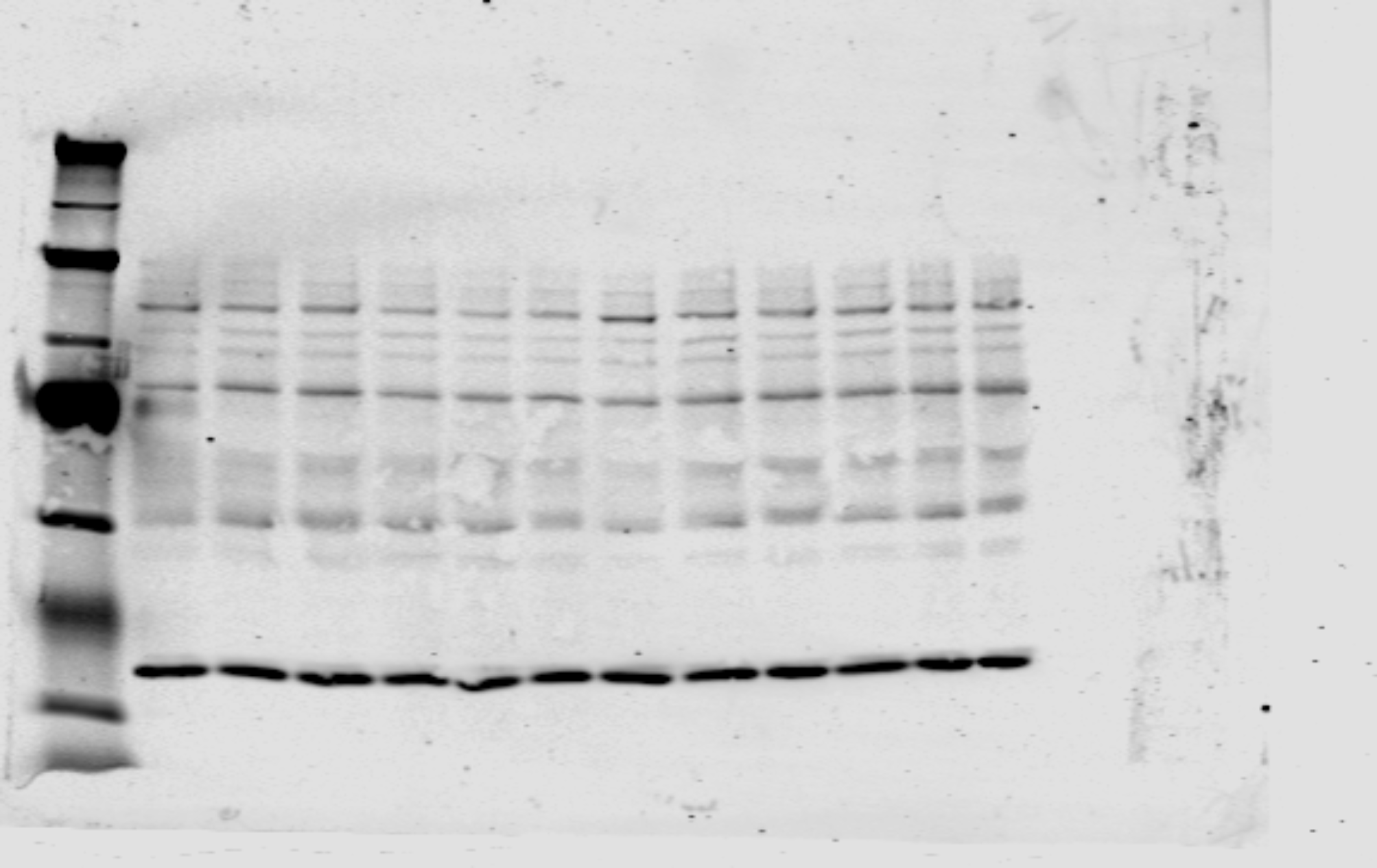

Supplement: Figure 3—source data 2. — Original membranes corresponding to Figure 3H and I. Paxillin, p-paxillin, Pyk2, and p-Pyk2 original membranes. [file elife-96810-fig3-data2.zip › tot Pyk2 uncropped blot.tif.tif]

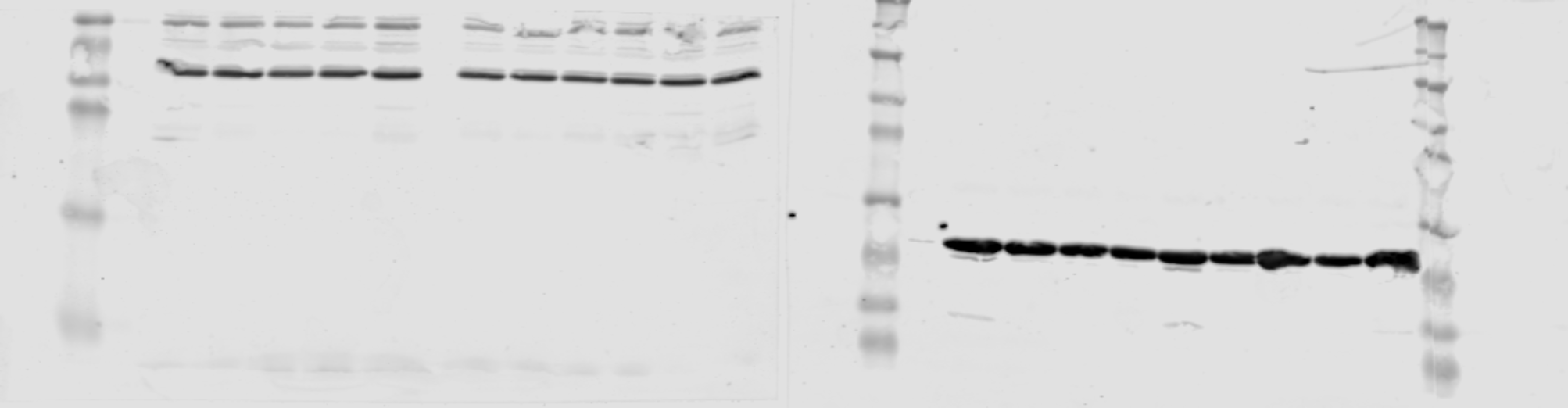

Supplement: Figure 4—figure supplement 1—source data 2. — Original membranes corresponding to Figure 4—figure supplement 1B and D. Calmodulin (CaM) and β-actin original membranes. [file elife-96810-fig4-figsupp1-data2.zip › B Actin.tif.tif.tif.tif]

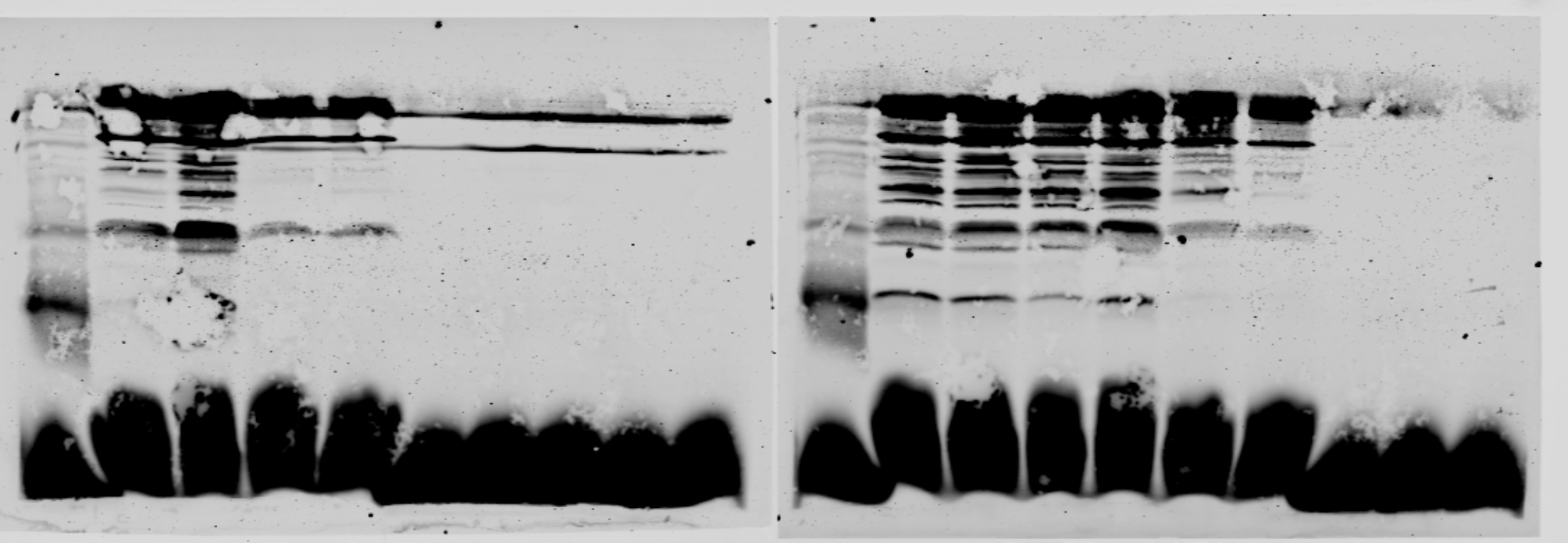

Supplement: Figure 4—figure supplement 1—source data 2. — Original membranes corresponding to Figure 4—figure supplement 1B and D. Calmodulin (CaM) and β-actin original membranes. [file elife-96810-fig4-figsupp1-data2.zip › CaM_GAPDH Lyz2xGCaMP5.tif.tif.tif.tif]

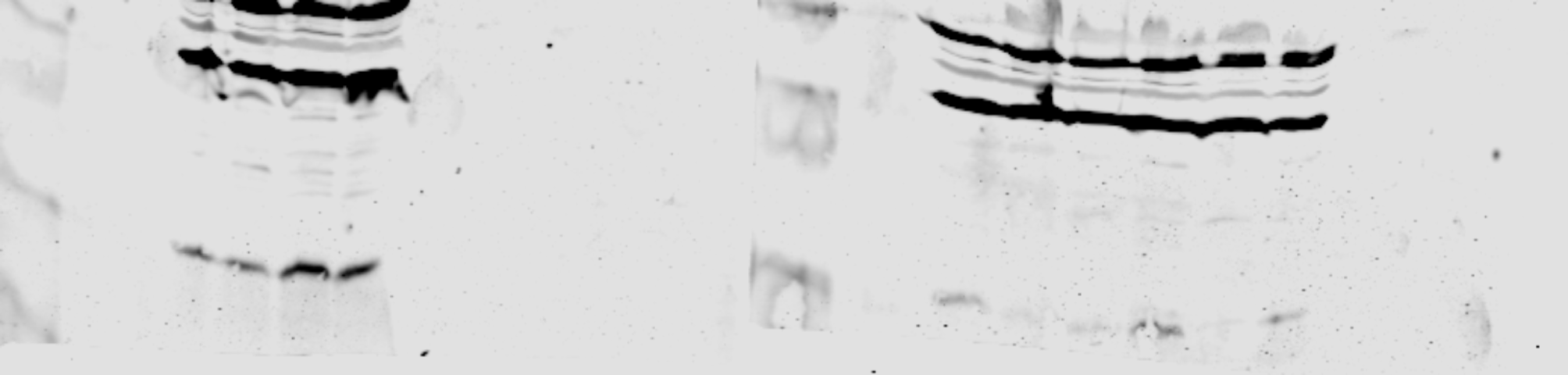

Supplement: Figure 4—figure supplement 1—source data 2. — Original membranes corresponding to Figure 4—figure supplement 1B and D. Calmodulin (CaM) and β-actin original membranes. [file elife-96810-fig4-figsupp1-data2.zip › CaM_GAPDH Lyz2xGCaMP5xS100a9.tif.tif.tif]
