# Supplementary material for: Cytosolic S100A8/A9 promotes Ca2+ supply at LFA-1 adhesion clusters during neutrophil recruitment
Source: eLife. 2024 Dec 19;13:RP96810. doi: 10.7554/eLife.96810 (PMC11658764; doi:10.7554/eLife.96810)
Supplement: Figure 4—figure supplement 1—source data 1. — Original membranes corresponding to Figure 4—figure supplement 1B and D. Calmodulin (CaM), GAPDH, and β-actin membranes are depicted and representative blots were then cropped and edited. Rectangle boxes indicate the representative bands used in the figure. Chamaleon Duo Pre-stained Protein Ladder was used as molecular weight marker. [file elife-96810-fig4-figsupp1-data1.zip › CaM_B actin WB.pdf]

CaM + GAPDH - *Lyz2xGCaMP5*

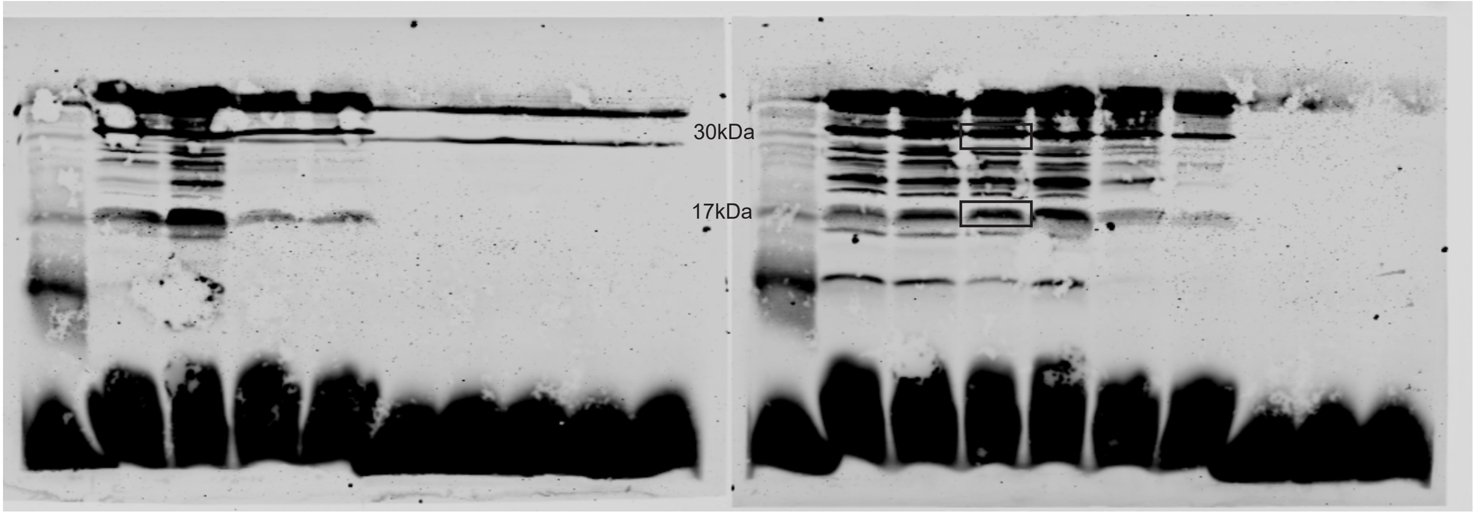

CaM + GAPDH - *Lyz2xGCaMP5xS100a9*$^{-/-}$

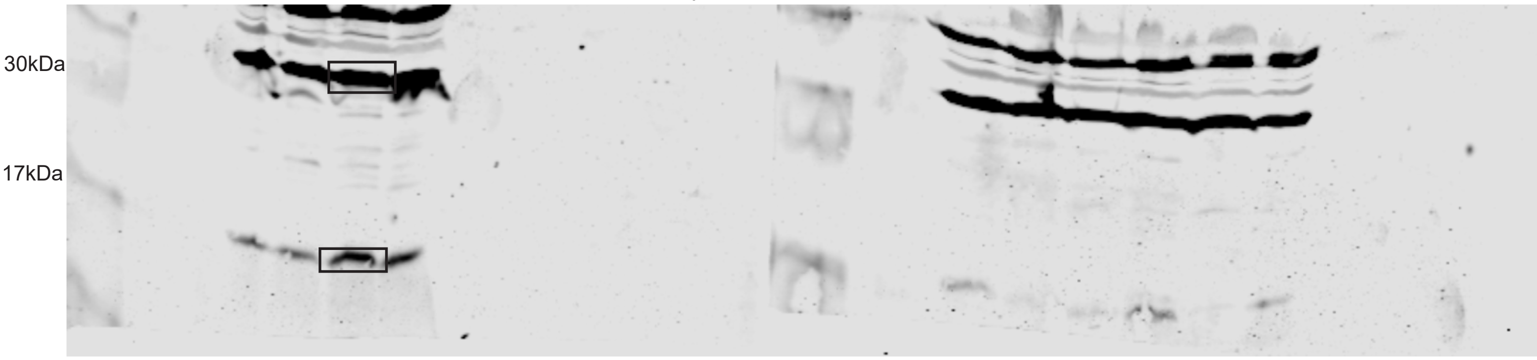

β-Actin

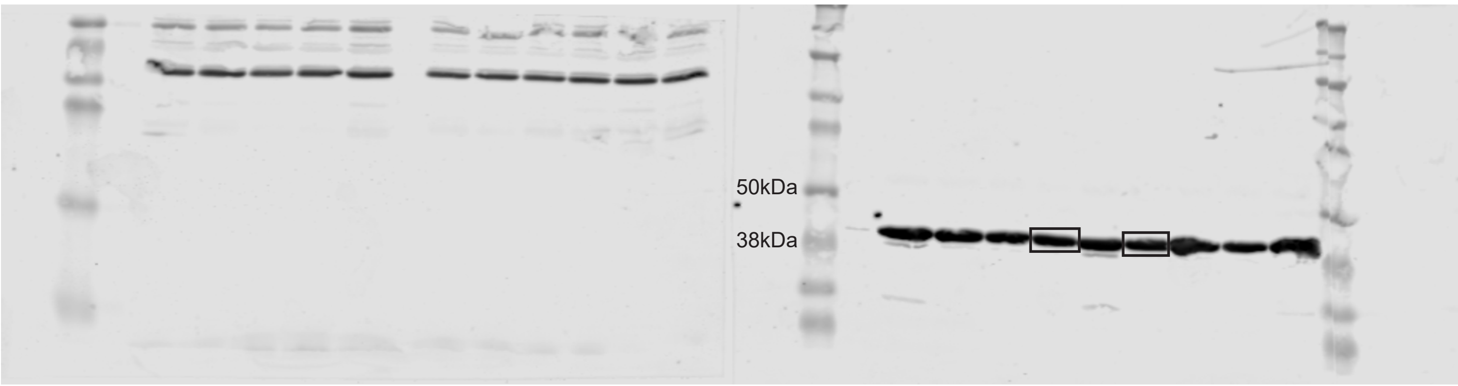

Figure 4-figure supplement 1-source data 1. Original Membranes corresponding to Figure 4-figure supplement 1, panel B and D. Calmodulin (CaM), GAPDH and β-Actin membranes are depicted and representative blots where then cropped and edited. Chamaleon Duo Pre-stained Protein Ladder was used as molecular weight marker.
